# Exochelin Production in *Mycobacterium neoaurum*

**DOI:** 10.3390/ijms10010345

**Published:** 2009-01-20

**Authors:** Kok-Gan Chan

**Affiliations:** Division of Genetics and Molecular Biology, Institute of Biological Sciences, Faculty of Science, University of Malaya, 50603 Kuala Lumpur, Malaysia and Centre for Research in Biotechnology for Agriculture (CEBAR), Institute of Postgraduate Studies, University of Malaya, 50603 Kuala Lumpur, Malaysia; E-Mail: kokgan@um.edu.my; Tel. +603-79675162; Fax: +603-79674509

**Keywords:** *Mycobacterium neoaurum*, siderophore, exochelin, iron-deficiency, ferriexochelin, zinc-deficiency

## Abstract

*Mycobacterium neoaurum* is a soil saprophyte and obligate aerobic bacterium. This group of mycobacterium is relatively fast-growing. They form colonies on nutrient agar at 37ºC within 3 – 4 days. In natural soil habitats, bioavailability of iron is limited. To facilitate iron uptake, most mycobacteria produce siderophores. One example is exochelin, which is extracellular and water-soluble. In this report, the production of exochelin in *M. neoaurum* was induced in iron-deficiency, but repressed under ironsufficiency growth conditions. It is however not induced under zinc-deficiency growth conditions. The growth of this mycobacterium was correlated with exochelin secretion under iron-deficiency culture conditions. When *M. neoaurum* was grown in defined medium containing 0.04 μg Fe(III)/mL (final concentration), the production of exochelin reached a maximum and the corresponding cell growth was comparable to that under iron-sufficiency conditions. In this study, exochelin was purified from spent supernatant of *M. neoaurum* by semi-preparative chromatography. When saturated ferric chloride solution was added into the purified exochelin, a ferri-exochelin complex was formed. It is proposed that iron uptake in *M. neoaurum* is exochelin-mediated.

## 1. Introduction

Iron is an important element to many living cells, including bacteria. It acts as a biocatalyst for a broad spectrum of redox reactions due to its ability to exist in aqueous solution in two stable oxidation states, Fe(II) (ferrous iron) and Fe(III) (ferric iron). Iron bioavailability in soil habitats and plant surfaces has been reported to be low [1]. To overcome this problem in the natural environment, plant growth-promoting bacteria produce low-molecular-weight compounds called siderophores to acquire iron [2]. Some plant growth-promoting bacteria can also utilize iron from heterologous siderophores produced by neighboring microorganisms [2].

Siderophores can be classified into three major structural types, *viz.* catecholates, hydroxamates, or (α-hydroxy)carboxylates. Siderophores have high affinity towards Fe(III) [3]. In each type of siderophores mentioned above, deprotonation occurs to form an anionic bidentate ligand or the desferri form, which can form very stable and selective complexes with Fe(III).

The desferri-form of siderophore reacts with iron to form the ferri-complex that can be used by the microbial cells for iron uptake. For this purpose, microorganisms have to tightly regulate enzymes and transport systems that allow well coordinated siderophore biosynthesis, secretion, siderophoredelivered iron uptake, and iron release [3]. In laboratory culture media, production of siderophores by microorganisms grown under iron-sufficiency can be as low as 0.1% of that produced under irondeficiency conditions [4].

There are four different types of iron chelating molecules involved in iron acquisition in mycobacteria, namely salicylic acid, citric acid, mycobactin and exochelin. Mycobactins can be further characterised into secretory-form (exomycobactin), and cell-associated form (mycobactin) [5–6]. Exochelins are peptidic siderophores that possess high affinity for iron [6]. In the present report, exochelin refers to the peptide-type siderophore. Exochelin is primarily produced by saprophytic mycobacteria, while exomycobactin is synthesized by pathogenic mycobacteria as their extracellular siderophore [7]. Some of the plant growth-promoting bacteria produce siderophores to competitively sequester the limited supply of iron available in the rhizosphere, restricting pathogenic fungi growth by rendering iron unavailable to them [8]. This report aims to examine the production of extracellular form of siderophore i.e. exochelin in *M. neoaurum* which may be used to elucidate the mechanism of biocontrol of plant pathogens.

## 2. Results

### 2.1. Production of exochelin in iron-deficiency, zinc-deficiency and iron-sufficiency conditions

All glassware used was treated using the previously reported method to remove traces of iron [9]. Growth medium termed l-asparagine medium, supplemented with various iron concentrations, was used for growth of mycobacteria. *M. neoaurum* formed golden or orange pigments when grown in this medium. Glucose instead of glycerol was used as carbon source as the former yielded a less viscous growth medium which rendered exochelin extraction easier.

**Figure 1.**
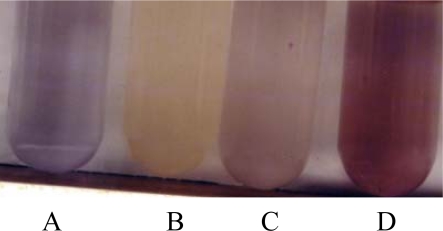
Detection of *M. neoaurum* exochelin in spent supernatants of *M. neoaurum* grown in iron-deficiency, zinc-deficiency and iron-sufficiency. CAS assay of exochelin in spent supernatant of *M. neoaurum* grown in iron-deficiency (B), zincdeficiency (C) and iron-sufficiency (D) conditions as described in the Experimental Section (4.3). Control experiment (A) was carried out by mixing the uninoculated medium with CAS solution. Formation of yellow solution in CAS assay indicates presence of exochelin.

*M. neoaurum* was cultured under iron-deficiency, zinc-deficiency and iron sufficiency conditions and its exochelin production assayed. Figure 1 illustrates the positive effect of iron-deficiency (0.02 μg Fe(III)/mL) on the production of exochelin in *M. neoaurum*. No detectable exochelin was observed in the spent supernatants obtained from both iron-sufficiency and zinc-deficiency conditions.

**Figure 2.**
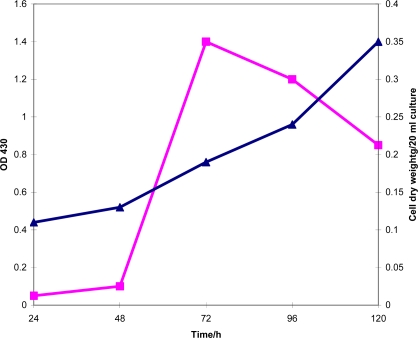
Time course analysis of *M. neoaurum* exochelin production and cell dry weight. Production of exochelin by *M. neoaurum* cells grown under iron-deficiency conditions (0.02 μg Fe(III)/mL). Cell growth was monitored from 24 to 120 h. Exochelin was concentrated by freezedrying the spent supernatant and was purified as described in the Experimental Section (4.2). Detection of exochelin production was measured by monitoring absorbance at OD 430 nm (▪). Cell dry weight (▴) (in g/20 mL) was measured by drying 20 mL cell culture at 90 ºC.

To determine the appropriate incubation time for maximum exochelin production in l-asparagine medium when grown under iron-deficiency (0.02 μg Fe(III)/mL) conditions, *M. neoaurum* cells were incubated at 37 ºC up to 120 h. Under this condition, exochelin production accelerates during active cell growth (within 24 to 48 h) and achieved the highest (OD_430_ >1.3) when incubated for 72 h with vigorous aeration (Figure 2). Exochelin concentration decreases gradually after 72 h (Figure 2).

### 2.2. Production of exochelin in media containing various concentrations of Fe(III)

**Figure 3.**
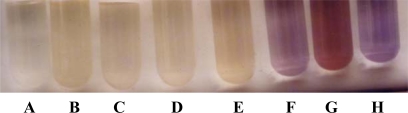
Detection of *M. neoaurum* exochelin in spent supernatants of *M. neoaurum* grown in various iron concentrations. Production of exochelin by *M. neoaurum* cells grown in various iron concentrations conditions (in μg Fe(III) /mL): 0.01 (A), 0.02 (B), 0.03 (C), 0.04 (D), 0.05 (E), 0.06 (F), and 1.0 (G). Exochelin was detected using CAS assay as described in Experimental Section (4.3). Formation of yellow solution indicates presence of exochelin. Control experiment (H) was carried out by mixing the uninoculated medium with CAS solution.

**Figure 4.**
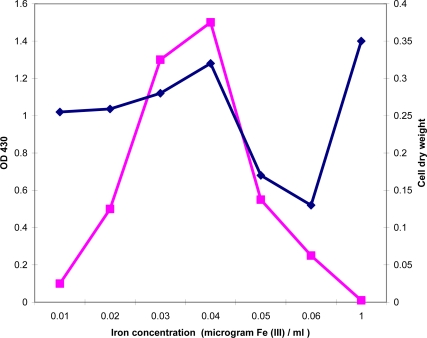
Detection of *M. neoaurum* exochelin production and corresponding cell dry weight in growth media containing various iron concentrations. Production of exochelin by growing *M. neoaurum* cells at various concentration of Fe(III) at 37 ºC for 72 h. Exochelin was concentrated by freeze-drying the spent supernatant and was purified as described in the Experimental Section (4.2). Detection of exochelin was measured by monitoring absorbance at OD 430 nm (▪). Cell dry weight (♦) (in g/20 mL culture) was measured by drying 20 mL cell culture at 90ºC as described in the Experimental Section (4.4).

To determine the optimal iron concentration for the maximal exochelin production in *M. neoaurum*, mycobacterial cells were grown in media containing iron concentration up to 1.0 μg Fe(III)/mL. No exochelin was detected in spent supernatant obtained from growth medium containing more than 0.06 μg Fe(III)/mL. This result suggests that 0.06 μg Fe(III)/mL is the threshold iron concentration for the induction of exochelin production (Figure 3). To obtain quantitative data, spent supernatants were assayed by spectrophotometric method. Interestingly, when *M. neoaurum* cells were grown in medium containing 0.04 μg Fe(III)/mL, the production of exochelin was the highest (OD_430_ ∼1.5), concomitant to a high mycobacterial cell growth which is comparable to that of iron-sufficiency condition (1.0 μg Fe(III)/mL) (Figure 4).

## 3. Discussion

It is difficult for microorganisms to obtain iron from the surrounding environment due to the extremely low aqueous solubility of ferric ion. Therefore, microorganisms have devised many strategies to acquire adequate soluble iron for aerobic growth. One of these strategies is to produce iron-binding compounds such as exochelin [6]. Herein secretion of exochelin by *M. neoaurum* into the medium during active growth under iron-deficiency conditions is reported. There was no indication that exochelin was metabolised during stationary phase [10]. It was also confirmed that *M. neoaurum* produced exochelin when grown under iron-deficiency (0.02 μg /mL Fe(III)) conditions, but not under iron-sufficiency and zinc-deficiency conditions. These data are in good agreement with previous findings [11].

When *M. neoaurum* cells were cultured under iron-deficiency conditions, exochelin was produced rapidly, followed by active cell growth, although it has been estimated that 7 to 64 μg of Fe(III)/g of mycobacterial cell mass is required to support growth [12]. Iron availability below these levels *in vitro* can result in poor cell growth in many species of mycobacteria. Intriguingly, *M. neoaurum* cells achieved the highest cell density after incubation for 72 h at 37ºC under iron-deficiency conditions. The trace amount of iron available in soil habitats and on plant surfaces compels soil microorganisms to compete fiercely for iron acquisition [13]. Since *M. neoaurum* is a natural soil saprophyte, this work may help understand the survival and function of plant growth promoting bacteria in soil where iron availability is low.

Disappearance of exochelin from the culture filtrate after prolonged incubation (>72 h) is in agreement with the previously reported work using *M. bovis* BCG [14] and *M. intracellulare* [15] as the test microorganisms. Lewin has reported that siderophore synthesis is tightly regulated by the amount of iron in the environment [16]. Since siderophores are involved in the acquisition of iron under iron-limiting conditions, the production of siderophores has been shown to be associated with virulence as well as the survival of many microorganisms [17].

It is suggested that mycobacteria are able to either solubilise or acquire iron in aqueous solution from the surrounding environment. This process is normally mediated by secretion of iron-binding compounds such as mycobactin, salicylic acid and exochelin [6, 9]. Here it is reported that *M. neoaurum* produced a water-soluble exochelin which is relatively stable in the present experimental condition. The data also suggest that production of exochelin may facilitate mycobacterial iron uptake and enhance cell growth in iron-deficiency condition.

It was also shown that exochelin production in *M. neoaurum* was the highest (OD_430_ ∼ 1.5) when grown in 0.04 μg /mL Fe(III). No exochelin was detected when *M. neoaurum* cells were grown in l asparagine medium containing more than 0.06 μg/mL Fe(III). This result suggests that exochelin production in *M. neoaurum* is repressible in the presence of abundant iron during growth. Mycobacterial cells may conserve energy by inactivating biosynthesis of exochelin when grown in iron-sufficiency condition. It is postulated that when the mycobacteria colonise soil habitats, it encounters iron-deficiency which in turn, triggers activation of exochelin biosynthesis for iron sequestration.

Surprisingly, when *M. neoaurum* cells were grown in iron-deficiency condition, their cell growth is comparable to that of iron-sufficiency condition (Figure 4). This result demonstrates clearly the role of exochelin in promoting cell viability and cell growth in iron-deficiency condition. It has been reported that bacterial siderophores help limit growth of pathogenic fungi through sequestration of iron in rhizosphere, rendering it a limiting nutrient for pathogenic fungi [18] and induced resistance in plants [19]. The present report may provide an insight on the attenuation of pathogenic soil fungi, either by supplementing exochelin to restrict iron bioavailability to fungi, or using siderophores-producing plant growth-promoting bacteria as a biocontrol for plants.

## 4. Experimental Section

### 4.1. Bacterial strains, growth media and culture conditions

The strain used in this study was *M. neoaurum* NCTC 10439 (a soil saprophyte). All glassware was pre-treated with potassium hydroxide to remove trace metals and especially iron, as previously described [9]. *M. neoaurum* culture was grown in l-asparagine medium that consists of (per 1000 mL of sterile deionised water) KH_2_PO_4_, l-asparagine and Al_2_O_3_ (0.5 % w/v each component). This medium was adjusted to pH 7.0, autoclaved and after cooling, filtered through Whatmann 541 filter paper. Approximately 900 mL of this medium was dispensed into tripled-baffled flasks and reautoclaved. After cooling, sterile d-glucose solution (10 % w/v) and trace elements (per 100 mL of medium) [MgSO_4_.7H_2_O (0.41 %, w/v), ZnCl_2_ (0.046 %, w/v), MnCl_2_ (0.01 %, w/v)] were aseptically added to sterile l-asparagine medium and in a final volume of 100 mL per flask. l-Asparagine medium was used as the iron-limiting medium for *M. neoaurum* with different iron concentrations by supplementing FeCl_3_ solution at the following final concentration (in μg Fe(III)/mL): 0.01, 0.02, 0.03, 0.04, 0.05, 0.06 and 1.0. Zinc-deficiency medium was prepared as described previously [20]. All cultures were incubated at 37 ºC for an appropriate time.

### 4.2. Purification and detection of exochelin

Seed culture of *M. neoaurum* was inoculated (1%, v/v) into liquid l-asparagine medium (1000 mL) containing up to 1.0 μg /mL FeCl_3_ and incubated with aeration at 37ºC for 72 h. Spent supernatant was collected by centrifugation at 5,000 × *g* for 15 min, and saturated FeCl_3_ solution was subsequently added dropwise until an orange complex began to form, and further stirred at 4ºC overnight. The orange precipitate was then removed by centrifugation at 5,000 × *g* for 15 min. A total of 300 mL of this clear, orange supernatant was transferred into a round bottom flask and rotary freeze-dried. The residue was reconstituted in sterile deionised water (40 mL). The solution was pipetted into a stirred ultrafiltration cell (Amicon Corp., U.S.A.) with YC05 membrane filter (Diaflo^®^). The operating pressure was set at 55 psi, with maximum of 70 psi. The ultrafiltration process was carried out at 4 ºC for 12 h.

Filtrate was collected in a sterile tube and subject to a high-resolving sulphonic acid ion-exchange resin (Bio-Rad AG50W-X4, 200–400 mesh, NH_4_^+^ form, 40 × 20 cm) at a flow rate of 1 mL/min. The column was washed with deionised water (50 mL) and the concentrated exochelin was eluted with a linear gradient of 0.1 M to 1 M NH_4_OH/acetic acid, at pH 6.0 and pH 9.0, respectively in a gradient mixer (Bio-Rad, USA), and monitored at 430 nm.

A total of 10 fractions was collected. The fractions with main peak were pooled to a final volume of 10 mL and desalted by gel filtration through Sephadex G10 (48 × 3 cm). The column was washed with deionised water (50 mL) and the red solution was loaded slowly into the column. Elution was done by adding deionised water at 2 mL/min. Five fractions were collected as the red solution began to elute.

The conductivity of each fraction was monitored using conductivity meter (Hanna, HI8033). The salinity of ultrapure water which gave 0 ppm CaCO_3_ and 0 μS of conductivity at 20ºC was referred to as standard. The volume of this salt-free fraction, as indicated by conductivity, was approximately 4 mL, and was stored at 4ºC. Spectrophotometry analysis of exochelin was carried out to determine its spectrophotometric properties at 430 nm. Estimation of exochelin production was carried out as reported [10].

### 4.3. Chrome azural S assay

For Chrome azural S (CAS) assay, it was carried out as reported previously [21] with modification. Briefly, cell-free spent supernatant (10 mL) obtained from cultures grown with different concentrations of Fe (III) and under zinc-deficiency conditions were individually mixed with CAS solutions. A change of the solution colour from blue to yellow indicated production of exochelin and the result was digitally recorded.

### 4.4. Estimation of cell dry weight

Dry mass of mycobacterial cells was obtained by drying 20 mL of cell cultures (grown at 37ºC for 72 h, normalised to OD_600_ 1.0) on a pre-weighed glass plate at 90 ºC.

## 5. Conclusions

To conclude, the results suggest that exochelin is necessary for good *M. neoaurum* growth and it may present an important aspect in the study of iron acquisition in soil habitats.
